# Barriers that Contribute to the Digital Divide Among Older Community Dwelling Adults in Later Life

**DOI:** 10.1093/geroni/igab046.3170

**Published:** 2021-12-17

**Authors:** Haowei Wang, Mai See Yang, Yong Kyung Choi

**Affiliations:** 1 The Pennsylvania State University, University Park, Pennsylvania, United States; 2 UC Davis, UC Davis, California, United States; 3 University of California Davis School of Medicine, Sacramento, California, United States

## Abstract

Research demonstrates that race and health literacy contribute to the digital divide, which is a major public health concern for older adults in the U.S. However, we still lack information about what types of barriers older adults have through a comprehensive examination using population -based data. This study focuses specifically on barriers to technology use among older adults. We use data from the Health and Retirement study 2012 Module “Technology Use: Barriers and Benefits” (N = 1,416). About 42% of participatnts did not use any technology (e.g., emails, social media, smart phone) (n = 501). The mean age for this non-user group was 72 years old (SD 10.3). 13% were foreign born, over half were female (56%), and the majory were somewhat educated (72% with a high school education or lower). About 23% of non-users were self-reported black, 16% Hispanic, 3% other race, and 58% non-Hispanic white. Barriers for adopting the use of technology included too difficult to keep up with the changes in technology (78%), too complicated (69%), not interested (65%), too much time required to learn (53%), too hard to learn (52%), expensive (43%), not easily available (24%), and opposed to using new technologies (27%). Results suggest that barriers were significantly correlated with more depressive symptoms among older adults who did not use technology. Compared to users, non-users were also more likely to have health conditions (e.g., hypertension, diabetes, lung disease, stroke, and arthritis). Findings of this study provide directions to address digital divide among older adults.

